# Growing Knowledge: Using Stem Cells to Study Developmental Neurotoxicity

**DOI:** 10.1289/ehp.118-a432

**Published:** 2010-10

**Authors:** Kellyn S. Betts

**Affiliations:** **Kellyn S. Betts** has written about environmental contaminants, hazards, and technology for solving environmental problems for publications including *EHP* and *Environmental Science & Technology* for more than a dozen years

A wealth of evidence attests that the organs of developing embryos, particularly the developing brain, are acutely sensitive to chemical perturbations. However, scientists know very little about how exposures to specific endogenous chemicals actually impact human development or children’s ability to learn. And there are almost no data on how the vast majority of the 84,000 chemicals currently listed in the Toxic Substances Control Act (TSCA) Inventory^1^—including most of the 201 compounds known to be neurotoxic to adults and the 1,000 chemicals shown to be neurotoxic to animals^2^—may affect developing infants. It is also unclear whether testing with animals always provides accurate insights into human developmental susceptibility.

A new line of research based on human stem cells is providing important insights into how chemicals may affect neonatal development. Stem cells are the master cells capable of producing some or all of the 200-plus different types of cells in the human body. In time, some researchers believe stem cells may enable scientists to amass far more data on how exposure to environmental chemicals affects human development, particularly the development of the brain. Now is a “critical time to be talking about stem cell research in the environmental health context,” says Tracey Woodruff, director of the Program on Reproductive Health and the Environment at the University of California, San Francisco (UCSF) Medical School.

## The Promise of Stem Cells

Most of the work now under way in the United States and the European Union (EU) does not use human embryonic stem cells, which a 23 August 2010 ruling by the Federal District Court for the District of Columbia said cannot be supported by federal funds.^3^ (On 9 September 2010, the U.S. Circuit Court of Appeals for the District of Columbia granted a request from the Justice Department that temporarily allows the government to resume funding research using human embryonic stem cells until the August decision is appealed.^4^) Instead, most of the projects aimed at expanding the ways to test for developmental neurotoxicity with human cells use neural stem cells—or neuroprogenitor cells, as they are sometimes called—derived from human fetuses.^5^

Neuroprogenitor cells are considered to be multipotent because they can give rise to the three major cell types of the human brain. The cells can be derived from fetal nervous system tissue, human embryos, or adult nervous system tissue, says Tim Shafer, a research toxicologist with the U.S. Environmental Protection Agency (EPA) Integrated Systems Toxicology Division.

Multipotent stem/progenitor cells are capable of generating some or all of the different types of cells required to maintain the health of one organ system. In contrast, pluripotent stem cells can produce the cells needed by more than one organ system. Embryonic stem cells are considered to be pluripotent. Only the zygote produced when a human sperm and egg merge and very early embryonic cells are truly totipotent in that they can generate any type of cell needed for human life including, importantly, the placenta and extraembryonic membranes (e.g., the amnion).^6^

The potential therapeutic applications of stem cells—such as for growing new skin for burn victims,^7^ aiding patients undergoing open-heart surgery,^8^ or producing brain cells to treat people with Parkinson disease^9^—have already received a great deal of attention. However, stem cells’ anticipated value as research tools may be even greater, according to experts at a workshop on the topic held in June 2010 by the National Research Council (NRC) Standing Committee on the Use of Emerging Science for Environmental Health Decisions.^10^

The ability of stem cells to differentiate into a wide variety of different cell types enables them to be used to model aspects of human biology that have been largely inaccessible to study by other means. In addition to prenatal developmental processes, this includes gene–environment interactions and the production of cell types that are difficult to maintain in the laboratory, such as liver, kidney, and nerve cells. In the past, scientists who wanted to study human neuronal tissue used cells derived from nervous system tumors, which “may not be normal cells,” Shafer explains. The other alternative was to “use animals to make the culture every time you want cells.”

Stem cells have the potential to improve how chemicals are evaluated because they involve using human cells, and they may be able to provide a broad range of data on a wide swath of chemicals much more quickly than conventional toxicology testing, Woodruff points out. “It’s a noninvasive technique for humans to test effects on humans,” she says.

Stem cells have the added advantage of obviating the need for laboratory animals. “There has been a push to decrease the use of animal testing,” Woodruff says, noting that such a reduction is an explicit goal of the European Union’s Registration, Evaluation, Authorisation and Restriction of Chemical Substances regulatory framework.

An NRC report released in 2007 pointed out that moving to *in vitro* methods, such as ones based on human stem cells, could greatly reduce the use of animals in testing, with the potential to eliminate animal testing altogether.^11^ Using conventional animal-based neurotoxicity screening tests to evaluate the tens of thousands of chemicals for which no neurotoxicity information is available “is just plain impractical,” says Shafer, who is involved in a project using neuroprogenitor cells to evaluate how chemicals may be affecting human neurodevelopment. “The cost would be too great, it would take years and years to accomplish, and . . . it would take literally millions of animals.”

Using human stem cells in toxicology testing can overcome other detriments of animal testing as well. Biologists have long suspected that because of differences between early embryonic development in rodents and humans, animal-based testing may not always be a good predictor of human developmental neurotoxicity, particularly in the earliest stages. “It could be that for some particular pathways [such as the ones mediated by the aryl hydrocarbon receptor (AhR)], there are going to be these species differences,” Woodruff says.

Differences between mouse cells and human cells “demonstrate that we do need to look at cells that are applicable to humans,” says Mary Alice Smith, an associate professor in the University of Georgia Environmental Health Science Department. “At this point, it looks as if there is a real difference between the human and the mouse cells for the AhR, and it may help explain some of the differences that we see between species.”

“Because signaling pathways do not develop simultaneously between species, toxicants can interfere in different ways,” explains Ellen Fritsche of the Environmental Health Research Institute in Düsseldorf and the University of Aachen. “Thus, animal experiments can over- or underestimate toxicities of chemicals—or give the right result. One must understand mechanisms of biological processes in testing species and in comparison with humans as well as the mechanisms of chemical toxicities to be able to understand the predictive value of animal experiments.”

Finally, Woodruff says, human cells may help scientists assess the incremental risks associated with the levels of exposure experienced by the general public. Using human cells also gives researchers the opportunity to begin to try to account for the human genetic variability and susceptibility based on different life stages and disease status.

## Research in 3-D

In Europe, teams in Germany and Poland are advancing the use of stem cells for developmental neurotoxicity testing. The first and only validated test for assessing developmental neurotoxicity, based on rodent embryonic stem cells, was developed at the European Commission’s European Center for the Validation of Alternative Methods.^12^ Now this group is working on implementation of human embryonic stem cells for developmental neurotoxicity testing. The Johns Hopkins University’s Center for Alternatives to Animal Testing is working with groups in Europe and the United States to coordinate how the new technologies for evaluating developmental neurotoxicity are implemented.

Fritsche heads up one of the leading teams from Germany. Her group has been using neurospheres, a commercially available three-dimensional (3-D) cell system created from human neuroprogenitor cells. Fritsche’s group has received funding from the German government to create neurosphere-based tests for assessing developmental neurotoxicity. The researchers have demonstrated that the cell systems can imitate some of the key processes of human neuronal development, including proliferation, differentiation, and migration.^13^ Efforts by researchers in Poland and at the U.S. EPA to create tests to assess developmental neurotoxicity using stem or neuroprogenitor cells are looking at similar end points.

Experts agree these are some of the processes critical to the formation of a functional nervous system.^14^ A carefully orchestrated symphony of enzymes, ion channels, and proteins all play important roles in the nervous system’s development, Shafer explains. In order for everything to develop properly, some processes must take place in a certain temporal sequence, whereas others are spatially dependent. If neurons cannot develop in the proper temporal and spatial sequence, or if the sequence is disrupted, the end result can be neurodevelopmental impairment, Shafer says.

The German researchers have demonstrated that exposing neurospheres to known neurodevelopmental toxicants including mercury chloride and methylmercury chloride decreases the number of nerve cells they produce and how far the cells migrate.^13^ Fritsche’s group also has used neurospheres to show exposure to polybrominated diphenyl ether (PBDE) flame retardants can alter human fetal brain cells by decreasing the distance that cells migrate by more than 25% during brain development.^15^ Most recently, they compared the responses of human and mouse neurospheres to polyaromatic hydrocarbons, whose developmental neurotoxicity is based on activation of the AhR. Fritsche and colleagues found the AhR was not activated in the human test cells and wrote that “an accumulating body of evidence now indicates that human AhR signaling is less operative than AhR function in most laboratory animals.”^16^

Neurospheres have the same weaknesses as other *in vitro* systems, Fritsche says: “No pharmacokinetics, as we do not have a whole organism, and limited metabolic capacity. The system is also limited to basic processes of brain development—no synaptic function, so far no network function, no higher cognitive functions to evaluate.”

But overall, neurospheres are very well suited for developmental neurotoxicity testing, Fritsche says. Neurospheres are the “right species,” Fritsche says, and they represent the interplay between the different neural cell types (e.g., protection of neurons and glial cells) and can produce test results much more quickly and less expensively than animal-based tests. Where conventional testing takes about 18 months and requires approximately 140 female animals and 1,000 offspring, the neurosphere assay takes four weeks (for assessing neuronal viability, proliferation, migration, differentiation, and apoptosis), Fritsche says. She estimates her group’s neurosphere-based tests cost approximately 80% less than conventional toxicology testing.

Fritsche contends the 3-D nature of the neurospheres also offers unique advantages. In unpublished work, she says she and her colleagues have shown that neurospheres express certain connexins, the molecules that form the gap junctions that mediate intercellular communication. This communication is known to play an important role in maintaining tissue health and responding to changes in the cellular environment. However, she admits she hasn’t yet attempted to study how similar the intercellular communication between neurospheres is to the way neurons communicate in the human body. She says she suspects “that at least some communication which exists *in vivo* is conserved after preparation of the cells.”

Another advantage of the neurospheres is that “cells are self-organized and influence each other by paracrine functions [which govern nearby cell signaling],” Fritsche says. “This makes the situation for the individual cell more ‘realistic’ than just a monolayer culture; [for example], some cells can protect neighboring cells.”

This is demonstrated by comparing tests conducted with PBDEs using neurospheres and a human neuroblastoma cell line, she says. Although concentrations of BDE-47 greater than 5 μM have been shown to kill human neuroblastoma cells,^17^ Fritsche says tests with human neurospheres showed no cytotoxicity at PBDE concentrations at least twice that high. However, the neurosphere tests did show that PBDEs had effects on migration and differentiation into neurons and glial cells.^15^ “We think it is important to investigate these end points as specific for developmental neurotoxicity in contrast to cytotoxicity, which is an unspecific measure,” Fritsche notes.

She and her colleagues have now evaluated how neurospheres are affected by exposure to seven chemicals, including polychlorinated biphenyls, benzo[*a*]pyrene, 3-methylcholanthrene, and 2,3,7,8-tetrachlorodibenzo-*p*-dioxin.^16^,^18^ They are currently assessing arsenic, the anticonvulsant valproic acid (which has been implicated in autism spectrum disorders^19^) and methylazoxymethanol (which is known to affect neural functioning in animals^20^) along with some other pesticides. To further validate the utility of neurospheres, the team also is testing them with compounds not expected to affect neural developmental, and their unpublished results suggest the assays can discriminate appropriately, she says. As a long-term vision, her group also plans to use progenitor cells from other organs to expand the kinds of toxicity testing they can do.

## Cutting the Cord… and Putting It to Work

More than a decade ago, scientists from the Polish Academy of Sciences led by Krystyna Domanska-Janik were inspired to find another source of human stem cells besides embryonic tissue because their country’s government forbids its use. In time, they succeeded in devising an uncontroversial source of pluripotent-like stem cells using human umbilical cord blood.^21^ From there, team member Leonora Buzanska, who now heads the Stem Cell Bioengineering Laboratory at the academy’s Mossakowski Medical Research Centre, found a way to generate a neural stem cell line.^22^

More recently, in collaboration with a research team led by Sandra Coecke of the European Commission Joint Research Centre’s *In Vitro* Models Unit, Buzanska and her Warsaw colleagues demonstrated this cell line can serve as the model system for developmental neurotoxicity testing.^23^ The group confirmed the line’s response to chemicals known to be neurotoxic, including sodium tellurite, methylmercury chloride, cadmium chloride, chlorpyrifos, and l-glutamate. The group also showed that the cells were not affected by exposure to acetaminophen, theophylline, or d-glutamate, and that their test system was able to identify differing susceptibilities in different developmental stages. For example, the tests revealed that the less differentiated cells were more sensitive to all of the known neurotoxicants except l-glutamate, which showed a higher toxicity to later stages.

Buzanska is currently collaborating with a team of researchers led by François Rossi of the European Commission Joint Research Centre’s Nanobiosciences Unit to develop what she calls a “microplatform for stem cell growth and differentiation with biofunctional microdomains.”^24^ The microdomains contain different “microenvironments” that enable the stem cells to differentiate until they reach one of a number of points of development, Buzanska explains.

The goal is to populate the platform with lineage-related stem cells that can be maintained at different developmental stages, beginning with the pluripotent stage and ending with functional neurons. The testing platform will enable scientists to simultaneously assess the influence of tested chemicals on different stages of neurodevelopment, as well as whether various microenvironments may play protective roles for the cells at defined stages, she says. Buzanska says her team is currently testing the microplatforms with methylmercury chloride. She hopes to publish her findings early in 2011.

Because processes of brain development are extremely complex, more than one *in vitro* model is needed to cover as many aspects as possible, Fritsche points out. “Buzanska’s cells could be a great addition to the proposed *in vitro* testing battery,” she says. “It would be extremely helpful to generate such umbilical stem cell lines from individuals representing human genetic diversity. The great advantage is that availability of umbilical cord blood is unlimited.”

## What the Future Holds

Both Buzanska and Fritsche believe their approaches to developmental neurotoxicity testing platforms will be speedier than conventional animal testing, but whether Buzanska’s platform will ultimately be capable of high-throughput testing is still an open question. Fritsche says neurospheres may not deliver more than what she calls “medium-throughput screening.” But not every compound has to be tested for developmental neurotoxicity, she points out; for instance, “the ones that don’t pass the placenta are not of interest [in this context].” The compounds that should be a priority for testing are endocrine disruptors and those that show some potential for developmental toxicity based on animal screening, she says. Therefore, even though it takes four weeks to complete developmental neurotoxicity testing with neurospheres, they could easily be used to test around 100 compounds per year with an automated setup, she says.

Shafer’s project under way at the EPA is following an approach similar to the ones initiated in Europe to use neuroprogenitor cells to evaluate how chemicals may affect human neurodevelopment. Shafer says his team’s goal is to develop what he calls a “first-tier” approach to identify chemicals that merit more detailed developmental neurotoxicity testing, perhaps following up with an alternative species model such as zebrafish and then a rodent or other mammalian species.

The neurodevelopmental processes for which Shafer and his colleagues are devising assays include proliferation, differentiation, neurite growth, the creation of synapse gaps (synaptogenesis), migration, myelination, and apoptosis. As the group works to develop assays to test how chemicals affect these processes, one objective is to ensure the assays are amenable to high-throughput testing, Shafer says. They are using monolayers of the commercial ReNcell CX model, derived from a human fetus. This will enable others to easily conduct the same tests, he says, and the two-dimensional monolayers also are more amenable to high-throughput testing than the 3-D neurospheres.

Of the neurotoxicity suite that Shafer’s group is developing, the proliferation assay is one of the furthest along. Neuroprogenitor proliferation is crucial to early brain development, when the neural tube expands rapidly and its anterior portion eventually gives rise to the brain. The proliferation assay initially proved itself in a small test with a group of chemicals documented to affect proliferation.^25^ More recently, the researchers tested the assay’s performance with the 309 biologically active chemicals being evaluated by the ToxCast™ program run by the EPA National Center for Computational Toxicology. The Shafer group’s assay showed that 125 ToxCast chemicals had a significant impact on neural proliferation.^26^ (Further unpublished work put that number at 112.) Participation in the program will allow these results to be compared with those from other screening and testing efforts, Shafer says.

At press time it was unclear how the ultimate fate of the August ruling on stem cells would affect projects of some of the researchers interviewed for this article. For instance, Smith is collaborating with Steve Stice, director of the University of Georgia Regenerative Bioscience Center, to develop a way to use “germ-like” cells derived from human embryonic stem cells to produce an *in vitro* system for investigating the developmental and reproductive effects of compounds that affect endocrine-system functioning. Early in human development, Smith explains, cells that eventually become part of the male or female reproductive system express two proteins, DDX4 and POU5f1. Smith and Stice had been measuring changes in metabolite levels in cells that express these proteins to determine the biochemical consequences of exposure to environmental chemicals. Smith says in time they also would like to look at impacts on neuronal cells.

In other human embryonic stem cell work, Woodruff and Mike McMaster of the UCSF departments of Cell and Tissue Biology and Obstetrics, Gynecology, and Reproductive Sciences are exposing stem cells to bisphenol A at levels similar to those measured in pregnant women and their fetuses. The team will examine how these exposures affect the cells’ gene expression profiles and developmental potential.

One possible alternative to human embryonic stem cells that was discussed at the June NRC meeting is what are known as induced pluripotent stem cells. In the past few years, scientists have begun to recognize that the somatic cells that compose the vast majority of the adult human body can be “reprogrammed” to create these induced pluripotent stem cells, says M. William Lensch of Harvard University’s Children’s Hospital and the Harvard Stem Cell Institute. This is possible because all of an individual’s cells contain the same DNA required to produce the entire body, he explains. An individual’s functional cells, such as skin cells and liver cells, differ from one another because they express different parts of the DNA. The reprogramming process involves the cells’ chromatin, which controls which genes are expressed.

Induced pluripotent stem cells are being used to produce a variety of human cell types, and researchers believe that, like embryonic stem cells, they can give rise to all of the body’s cell types. In the future, human induced pluripotent cells may prove useful for developmental neurotoxicity testing, Shafer says. They have the potential to help researchers represent a greater degree of genetic variability, he says.

## The Regulatory Imperative

Until recently, most of what was known about developmental neurotoxicity was the result of tragic accidents with lead, methylmercury, polychlorinated biphenyls (PCBs), arsenic, and toluene.^2^ In the past few years, prospective birth cohort epidemiologic studies undertaken in the NIEHS-funded Centers for Children’s Environmental Health and Disease Prevention Research have taught us more, indicating that arsenic, manganese, organochlorine pesticides, polybrominated diphenyl ethers, phthalates, and bisphenol A also are likely to adversely affect infant brain development.^2^

Philip Landrigan of the Mount Sinai School of Medicine points out that of the approximately 3,000 chemicals used in quantities of more than 1 million pounds per year in the United States, only a small fraction have been tested at all to assess whether they can cause damage to the developing brain and nervous system. “Eighty percent of the chemicals to which children are exposed every day from conception on are chemicals whose possible toxic effects on the brain and nervous system are simply unknown,” he says.

But neurobehavioral damage caused by environmental chemicals is preventable, and in an influential 2006 paper, Landrigan and Philippe Grandjean of the Harvard School of Public Health argued that “testing of new chemicals before allowing them to be marketed is a highly efficient means to prevent toxicity.”^2^ At the end of the day, Shafer says, “anything that can be done to increase the number of chemicals that we can collect toxicological information on is an improvement [over the current state of affairs].”

However, having good tests in place to conduct accurate hazard assessments of the chemicals in use today is only part of the solution. Without a legislative or regulatory mandate to conduct such testing, public health experts like Landrigan question whether it will be done. He says he blames the chemical industry for not taking responsibility for the products they make and sell, and he faults the government for not enforcing the law.

“I’m very frustrated that the testing has just not been done for so many of these chemicals. I think the failure to test chemicals under [TSCA] represents a grave lapse in stewardship,” Landrigan charges. Bills that would revamp TSCA and require more chemical testing have been introduced in both houses of the U.S. Congress.^27^,^28^ Additionally, the EPA has proposed several rules intended to improve chemical reporting under TSCA.^29^

Meanwhile, people today, and especially children, are exposed to thousands of chemicals that have never been properly tested for toxicity—or tested at all, Landrigan says. “It is a very worrisome prospect,” he says, “to consider that chemicals that children are exposed to every day that are widely detected in the blood of pregnant women, in breast milk, and in cord blood could well be neurotoxic chemicals whose neurotoxicity has just never been properly examined.”

**Using Stem Cells to Investigate Autism**

Ricardo Dolmetsch, a neurobiologist at Stanford University, is employing a very different approach to using stem cells in research related to children’s environmental health. Dolmetsch’s group is using human stem cells to investigate autism spectrum disorders (ASDs), and his group is laying the groundwork that may eventually allow them to create a system for screening environmental agents to assess whether they could play a role in these disorders, he says.

The reported incidence of ASDs has been rising, and although some circumstantial evidence points to environmental agents, good data are sorely lacking, Dolmetsch says. Tracking down links with conventional animal models is problematic because scientists believe some important classes of neurons exist only in primates, he points out.

Dolmetsch has succeeded in identifying some of the genes involved in ASDs by focusing on relatively rare disorders known to have a genetic link. Such groups of patients are likely to have similar “cellular molecular signatures,” he explains.

He and his colleagues harvest cells from these patients’ skin and reprogram them to become induced pluripotent stem cells, which are then converted into functional cells. The research team has successfully produced a wide variety of neurons and glial cells, including catecholaminergic cells, which control the secretion of hormones such as dopamine that have been implicated in both schizophrenia and autism.

In unpublished work presented at the NRC meeting in June,^10^ Dolmetsch’s group reported success at identifying genes associated with the calcium signaling deficits and cardiac irregularities found in patients with Timothy syndrome, a rare disease characterized by webbed fingers and toes that is often associated with developmental delays and autism. They have also discovered a compound that appears to reverse the calcium signaling issues, which Dolmetsch says may ultimately help pharmacologists discover a drug that would help children with the syndrome.

The Stanford group is investigating links their work has turned up with other rare ASDs, too. They credit their successes thus far to what Dolmetsch calls the focused approach of narrowing in on genes they suspect to be involved with the diseases they are studying. Although Dolmetsch’s group has not yet used induced pluripotent stem cells to study environmental triggers of ASDs, he says he believes “that at least in principle it is doable.”

## Figures and Tables

**Figure:**
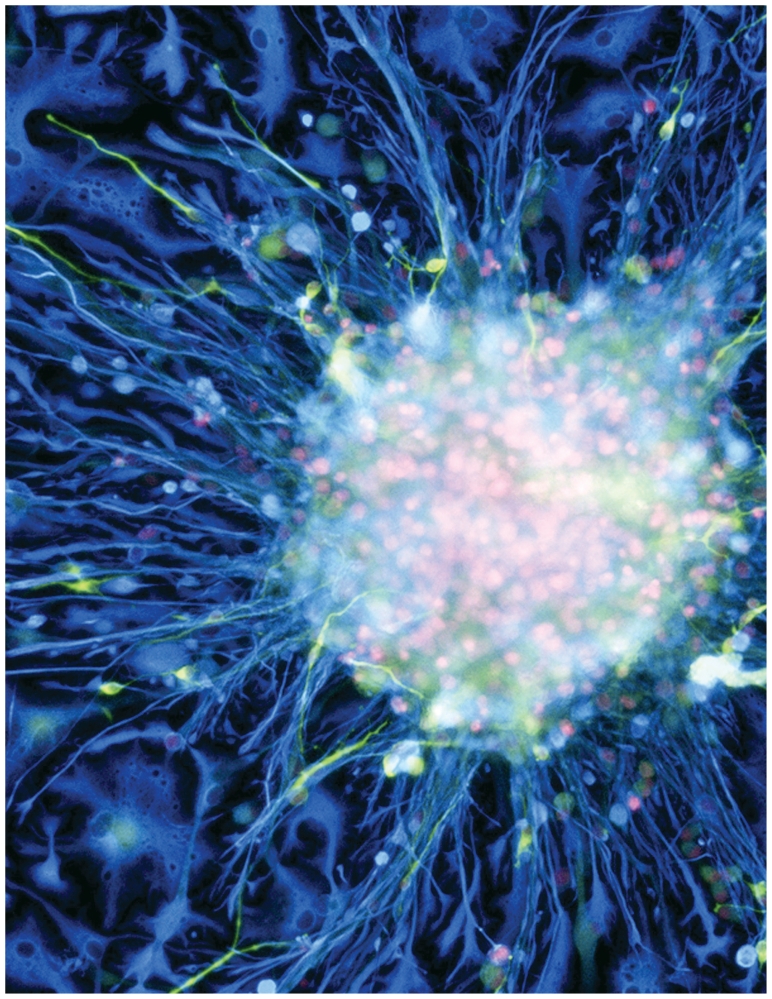
Neural stem cell culture Fluorescent light micrograph of a cluster of human neural stem cells shows the cells differentiating into different cell types as they migrate out from the central neurosphere. Lighter blue indicates astroglial fibrillary acidic protein, yellow indicates neuronal tubulin III, and pink indicates nuclear DNA.

**Figure:**
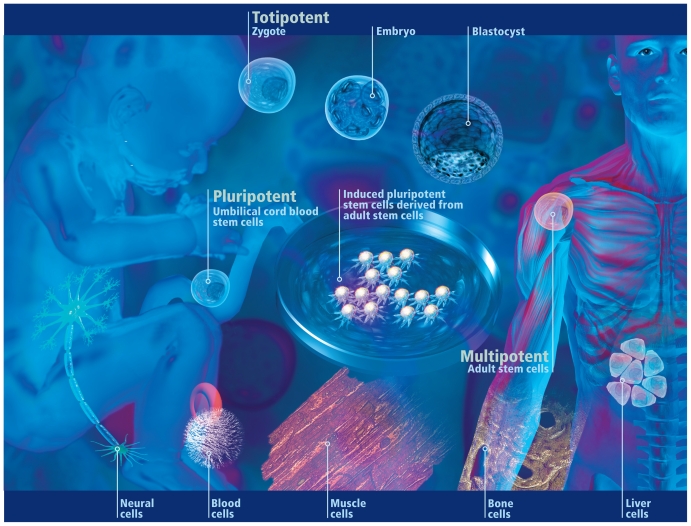
Stem cells: a potent force Potency denotes a stem cell’s potential to differentiate into different cell types. Totipotent stem cells produced from the fusion of an egg and sperm cell can differentiate into any cell type including the placenta and extraembryonic membranes. Pluripotent stem cells can differentiate into nearly all cell types. Multipotent stem cells—undifferentiated cells found in many tissues of the adult body—can differentiate into a number of cells within a single organ system.
